# Transfer Accuracy of Closed‐Tray Implant Impressions: An In Vitro Comparison Across Five Implant Systems

**DOI:** 10.1002/cre2.70394

**Published:** 2026-06-23

**Authors:** Walter Zweigardt, Benedikt Schneider, Dragan Ströbele, Richard Mosch, Constantin von See

**Affiliations:** ^1^ Research Center for Digital Technologies in Dentistry and CAD/CAM, Department of Dentistry, Faculty of Medicine and Dentistry Danube Private University Krems an der Donau Lower Austria Austria; ^2^ Center for Oral and Maxillofacial Surgery, Department of Dentistry, Faculty of Medicine and Dentistry Danube Private University Krems an der Donau Lower Austria Austria

## Abstract

**Objective:**

This in vitro study aimed to evaluate and compare the transfer accuracy of implant impression systems utilizing transfer caps for the closed‐tray technique across five different implant systems, with the open‐tray (pick‐up) technique serving as a reference.

**Materials and Methods:**

Six bone‐level implants from different manufacturers (SIC Invent, BEGO, Bredent, Camlog, and Straumann) were placed in a 3D‐printed custom‐designed master model. The master model with the inserted implants and the attached scan bodies served as the reference model. The implant master model was duplicated 10 times by means of conventional impressions. Based on these impressions, the master models were fabricated. Finally, closed‐tray impressions using transfer caps were taken ten times for each system, and one system (SIC Invent) was also tested using the open‐tray technique. Polyether impression material and standardized laboratory protocols were used throughout. The accuracy of the impressions was evaluated through 3D superimposition using Final Surface software, comparing master models to a reference model. Deviation measurements were statistically analyzed using rank‐based ANOVA.

**Results:**

No statistically significant differences were found in the transfer accuracy among the five closed‐tray systems. Similarly, the closed‐tray technique showed comparable precision to the open‐tray technique.

**Conclusion:**

Closed‐tray implant impressions using transfer caps can achieve transfer accuracy on par with the open‐tray technique. These findings support the potential clinical reliability and reproducibility of closed‐tray systems across multiple implant manufacturers.

## 1 Introduction

Dental implants have significantly expanded therapeutic possibilities in restorative dentistry, delivering functional and aesthetic results for a growing number of patients. Clinical outcomes in this field have substantially improved over the years, thanks to advancements in surgical techniques, materials science, and instruments (Schmidt et al. 2019).

To ensure continuous advancements, implant manufacturers invest significant resources in research and development. These investments form the foundation for the advancement of the entire field of dental implantology, as significant progress in dentistry and dental technology is closely linked to trends in materials research. Despite increasing digitization within dentistry, conventional implant impressions still remain an established method for the accurate and largely error‐free transfer of the three‐dimensional implant position from the patient's mouth to the plaster model. An exact and error‐free transfer of the clinical position of the implant to a working model ensures a precise fit of the prosthetic restoration, leading to a better long‐term outcome (Schmidt et al. 2019; Wöstmann et al. 2008). Implant impressions can be performed using a wide range of methods and materials. In daily practice, the open‐ and closed‐tray techniques are most commonly used.

Implant impressions can be performed using a wide range of methods and materials. In daily practice, the open‐ and closed‐tray techniques are most commonly used. The open‐impression technique, also referred to as the pick‐up technique or direct method, transfers the implant position via an undercut impression post, which ultimately remains in the impression. Therefore, the impression post is secured in the implant using a fixing screw and then positioning is taken up with an impression. The pick‐up technique can usually only be carried out using an individually manufactured or prefabricated spoon, as this must ensure that the retaining screw is loosened before removal from the patient's mouth (Flügge et al. 2018; Simeone et al. 2011).

The closed impression is also known as the reduction technique or indirect method. In contrast to the pick‐up technique, it transfers the implant position via a non‐undercut impression post, which initially remains connected with the implant after the impression has been taken. The transfer post is only removed from the implant after the tray has been removed, unscrewed, and then reduced into the impression, hence the term reduction technique. The indirect method is usually carried out with a ready‐made metal tray or a semi‐custom impression tray. The use of an individual tray is also possible, but not necessary. The challenge of closed impressions is to correctly reposition the impression post into the impression. This is a sensitive process, as transfer errors in the implant position can occur, possibly resulting in prosthetic failure (Schmidt et al. 2018; Moreira et al. 2015).

To combine the advantages of both techniques, some implant manufacturers have introduced transfer caps. In this approach, a transfer cap attaches to the screwed‐in impression post and remains in the impression material after removal, while the impression post stays connected to the implant. Subsequently, the impression post is detached from the implant and repositioned into the transfer cap within the impression. Some studies suggest that closed‐tray techniques using transfer caps may improve the precision of the conventional closed‐tray method under specific conditions. However, the current literature remains inconclusive, with several investigations reporting comparable or even superior accuracy for alternative impression approaches (Kwon et al. 2011; Nakhaei et al. 2015).

Both impression techniques are widely used in clinical practice. Although the closed‐tray technique offers simpler clinical handling, the open‐tray technique is often recommended for its purported superior accuracy. Numerous studies have explored the advantages and disadvantages of both techniques concerning their precision (Flügge et al. 2018).

This in vitro study aims to evaluate the transfer accuracy of implant impression systems using transfer caps from different manufacturers for the closed‐tray technique.

## 2 Materials and Methods

This in vitro study evaluated six different bone‐level implant systems and their respective closed‐impression systems (Table 1): One SIC Invent SICace (SIC Invent, Basel, Switzerland), one BEGO Semados (BEGO Implant Systems, Bremen, Germany), one Bredent copaSKY (Bredent Medical, Senden, Germany), one Camlog Progressive Line (Camlog Biotechnologies, Basel, Switzerland), and one Straumann Bone‐Level Regular CrossFit implant (Straumann Holding, Basel, Switzerland), along with their respective impression posts and transfer caps. For comparison with an open technique, a second SIC Invent SICase was used along with a corresponding impression post (Table 1).

**Table 1:** List of implants used.

Manufacturer	Implant type	Diameter	Length
SIC Invent (Basel, Switzerland)	SICace	4.0 mm	9.5 mm
BEGO Implant Systems (Bremen, Germany)	Semados S	4.1 mm	10 mm
Bredent Medical (Senden, Germany)	copaSKY	4.0 mm	10 mm
Camlog Biotechnologies (Basel, Switzerland)	CAMLOG PROGRESSIVE LINE	4.3 mm	9 mm
Straumann Holding (Basel, Switzerland)	Bone‐Level Implant SLActive	4.1 mm	10 mm

The setup and measurement of the models involved the use of Netfabb Premium 2021.1 (Autodesk, San Rafael, USA), DentalManager software (3Shape, Copenhagen, Denmark), the Asiga Pro 4 K 3D printer (Asiga, Alexandria, Australia), and Final Surface software (GFal, Berlin, Germany). The master models were created using laboratory analogs recommended by the manufacturers. Additionally, scan bodies were used in this study for creating a reference model and measuring the models. All impressions were made as monophase impressions exclusively with the 3 M™ Impregum™ Penta™ Soft Polyether (3 M Germany, Seefeld, Germany) impression material. Fujirock EP Premium Line super‐hard gypsum class IV (GC Europe, Leuven, Belgium) was used to produce the gypsum models.

A custom implant master model was created to compare the transfer accuracy of implant impressions. This model was designed using Netfabb software (Autodesk, San Rafael, USA). A maxillary premolar (tooth 25) was incorporated into the model, with the incorporated premolar serving as a spatial reference. The implants were then positioned parallel to each other at a distance of 8 mm, to simulate a clinical arch situation and to ensure standardized spatial orientation and implant positioning within an anatomical context.

**Figure 1:**
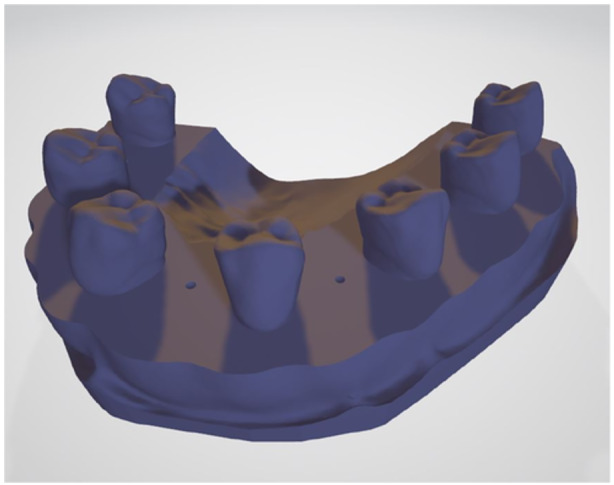
Constructed implant master model.

The spaces between the premolars served as placeholders for the implants, which had diameters between 4.0 and 4.3 mm, and maintained a minimum distance of 1.5 mm from the premolars during insertion; see Figure 1: Constructed Implant Master Model.

**Figure 2:**
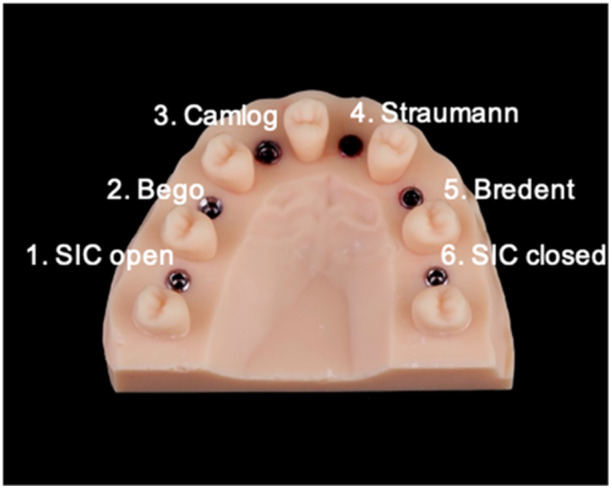
Manufactured implant master model.

The final design in Netfabb (Autodesk, San Rafael, USA) was printed using the Asiga Pro 4 K printer (Asiga, Alexandria, Australia). The last step involved implanting the implants into the 3D‐printed master model. First, the designated spaces between the premolars were drilled using the precision milling device model D‐F 44 (Harnisch + Rieth, Winterbach, Germany), matching the individual implant diameters. The six implants were then inserted into the drilled holes according to the manufacturers' guidelines and using the designated trays, as shown in Figure 2. The selected implants had diameters between 4.0 and 4.3 mm and lengths of 9 or 10 mm, and were positioned parallel to each other and to the premolars. The implants were inserted in the following sequence from left to right: 1. SIC Invent (open tray), 2. Bego, 3. Camlog, 4. Straumann, 5. Bredent, and 6. SIC Invent (implants 2–6: closed tray).

**Figure 3:**
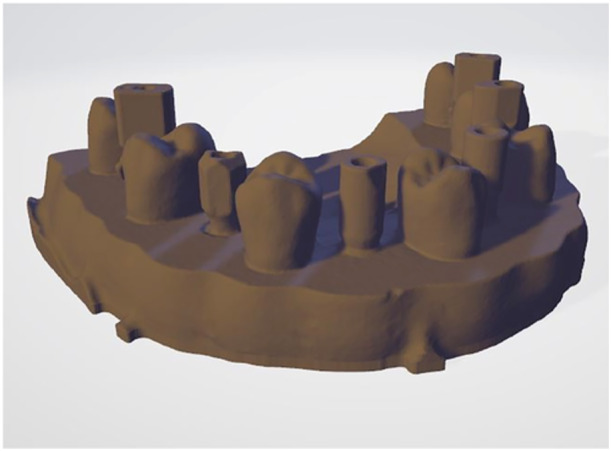
Manufactured reference model.

The printed implant master model, with the inserted implants and attached scan bodies, served as the reference model. The model with scan bodies was scanned using the 3Shape D800 lab scanner (3Shape, Copenhagen, Denmark) and the corresponding 3Shape software with a scan accuracy of < 1 µm. Figure 3 shows the reference model.

The implant master model was molded 10 times under standardized conditions in the university's laboratory. The number of impressions was based on the mean value from numerous existing studies related to the transfer accuracy of implant impressions. All impression posts were tightened using a standardized torque of 10 Ncm, in accordance with the manufacturers' recommendations.

**Figure 4:**
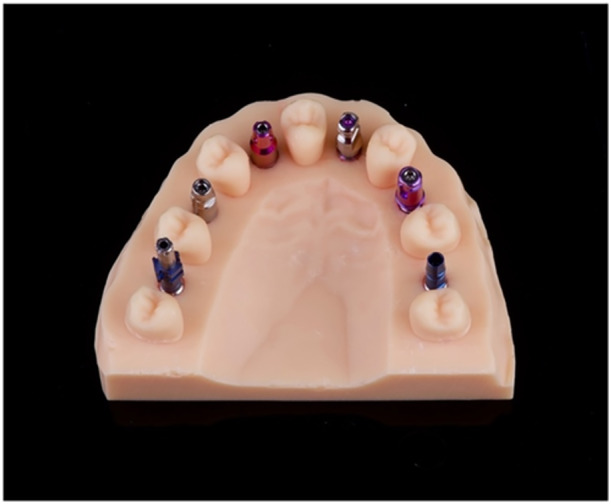
Implant master model including impression posts.

**Figure 5:**
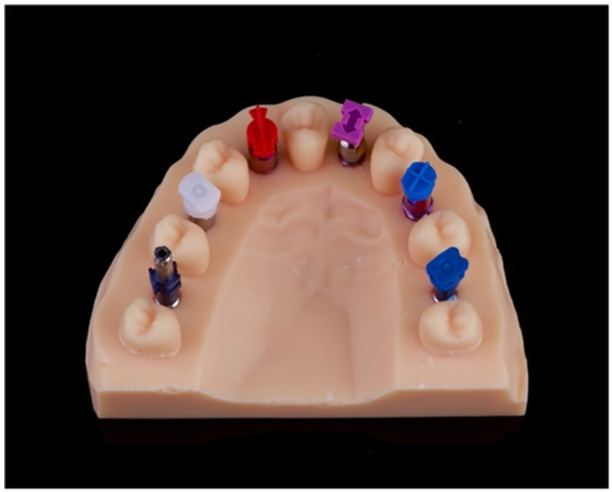
Implant master model including transfer caps.

The ambient temperature was maintained constant at 21°C (±1°C) with a humidity of 40% (±10%) for all steps of the experiment. Impressions were taken by an experienced operator using a standardized protocol to eliminate operator variability. For each impression, a prefabricated tray was used with standardized openings where necessary (e.g., for the open‐tray SIC Invent system). The impression trays were each filled with 3 M™ Impregum™ Penta™ Soft (3 M Germany, Seefeld, Germany). Mixing was performed automatically using the 3 M Pentamix™ 3 mixing device (3 M Germany, Seefeld, Germany). The polyether impression material set within 5 min. Finally, the impression was allowed to rest for 60 min to allow the polyether material to fully recover. It should also be noted that a new transfer post and transfer cap were used for each of the ten impressions, and each impression was taken with its own, individually printed impression tray. Figures 4 and 5 illustrate the implant master model with transfer setups and caps.

**Figure 6:**
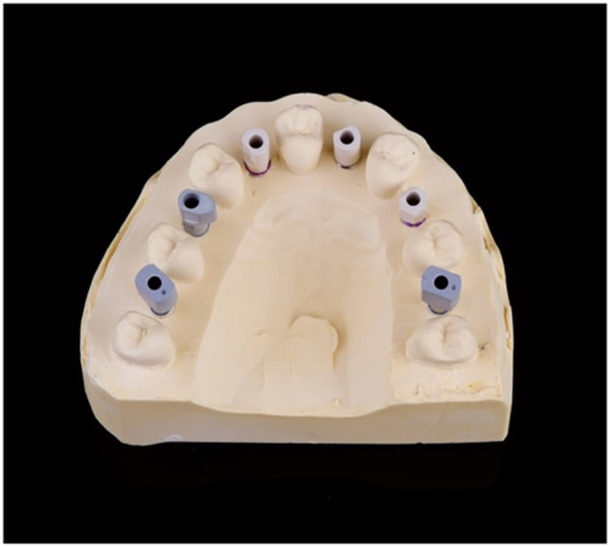
Manufactured master model including scan bodies.

The master models were created from the impressions of the implant master model. The transfer posts used in the impressions were repositioned onto the transfer caps in the tray, and the corresponding laboratory analogs were screwed onto the posts. The impressions were then cast with Fujirock EP Premium Line super‐hard gypsum class IV (GC Europe, Leuven, Belgium). The casts were removed from the tray after 90 min and stored for 10 days in a constant‐temperature room at 21°C (±1°C) with a humidity of 40% (±10%). Finally, the scan bodies used in the reference model were screwed onto the laboratory analogs in the master model, and a digital impression of the master model was taken with the 3Shape D800 lab scanner (3Shape, Copenhagen, Denmark). Figure 6 shows one of the master models equipped with scan bodies and scanned.

**Figure 7:**
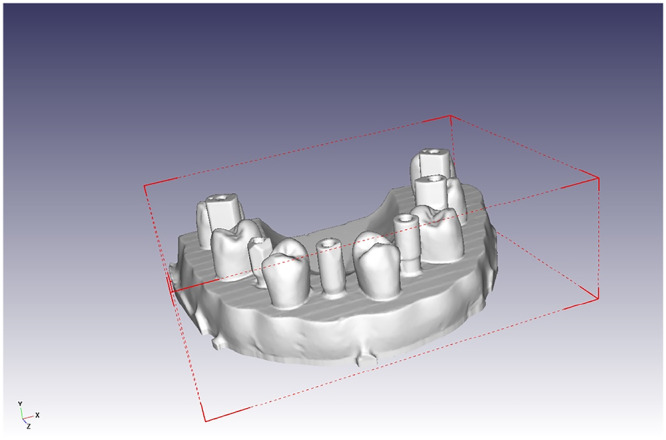
Scanned reference model in Final Surface.

**Figure 8:**
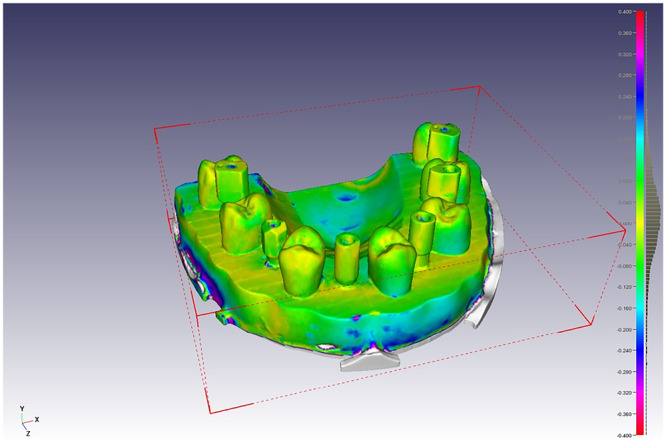
Overlay of master and reference models in Final Surface.

The digital measurement of the models was performed with Final Surface software 2022.0.2 (GFal, Berlin, Germany), which can overlay three‐dimensional objects, allowing for a comprehensive analysis of transfer discrepancies between master models and the reference model. Figures 7 and 8 show the models in Final Surface (GFal, Berlin, Germany).

The master models were aligned to the reference model in Final Surface (GFal, Berlin, Germany) using the modified best‐fit method, and then evaluated via distance analysis at three measurement points on the scan bodies: occlusally, buccally, and mesio‐distally. The mean value of the deviations for each scan body was calculated from the three measurements. Statistical analysis was performed using SigmaPlot 13.0. Data distribution was assessed descriptively. Individual master models (e.g., models 2 and 6) showed deviations from normal distribution due to the presence of outliers. Given the limited sample size and the exploratory nature of this in vitro study, a rank‐based analysis of variance (ANOVA) was applied. The level of significance was set at 5% (*p* ≤ 0.05). Results should therefore be interpreted with appropriate caution.

## 3 Results

To assess the results, the average deviation for each scan body was calculated from the three measurement points (see Table 2: Listing of mean values per scan body). The “SIC Invent closed” transfer setups showed a minimum deviation of 0.02 mm and a maximum deviation of 0.14 mm, whereas the “SIC Invent open” impression posts showed a minimum deviation of 0.05 mm and a maximum deviation of 0.19 mm.

**Table 2:** Mean values per scan body measured in millimeters (mm).

	1. SIC open	2. Bego	3. Camlog	4. Straumann	5. Bredent	6. SIC closed
Model 1	0.05	0.03	0.04	0.04	0.03	0.02
Model 2	0.06	0.05	0.05	0.06	0.26	0.05
Model 3	0.06	0.04	0.03	0.06	0.04	0.03
Model 4	0.05	0.04	0.03	0.02	0.03	0.06
Model 5	0.19	0.07	0.05	0.03	0.07	0.14
Model 6	0.14	0.01	0.02	0.03	0.02	0.07
Model 7	0.09	0.15	0.10	0.07	0.04	0.07
Model 8	0.05	0.03	0.03	0.02	0.01	0.05
Model 9	0.07	0.04	0.02	0.03	0.06	0.08
Model 10	0.12	0.09	0.05	0.04	0.03	0.05

**Figure 9:**
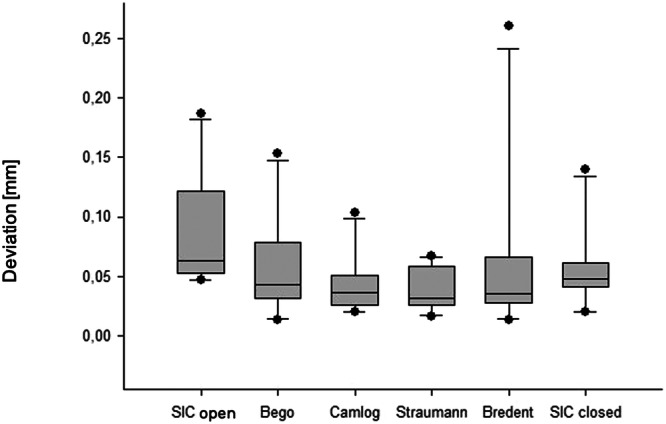
Boxplot: comparison of deviations.

Across all implant systems, isolated higher deviation values were observed in individual models, with occasional deviations exceeding the typical range of approximately 0.02–0.07 mm by a factor of three to five. These deviations were not confined to a single implant system or impression technique, but occurred sporadically across both open‐ and closed‐tray groups. The most pronounced deviation was observed in one Bredent model (0.26 mm), whereas increased values were also present in individual models of the SIC open, BEGO, CAMLOG, Straumann, and SIC closed groups. Importantly, no systematic pattern or consistent increase in deviation was identified for any specific implant system or impression technique. No significant differences in transfer accuracy were found when comparing the closed‐tray impressions among the five systems from SIC, Bego, Bredent, Camlog, and Straumann. Similarly, there were no significant differences when comparing the closed‐tray technique to the open‐impression posts (SIC‐invent, Basel, Switzerland). The box plot shown in Figure 9 visualizes the results.

## 4 Discussion

The literature describes various approaches to assessing the accuracy of implant impressions. Generally, measurement methods can be divided into direct and indirect procedures regarding the accuracy of the impressions. The direct procedure measures only the immediate dimensional change of the impression, omitting the creation of gypsum models. In contrast, the indirect measurement method involves using gypsum models, which can be implemented in both two‐ and three‐dimensional analyses. Most studies use the indirect method, as direct scanning of conventional impressions yields poorer results compared to scanning gypsum models (Güth et al. 2016; Ender and Mehl 2015; Lin et al. 2015; Ender and Mehl 2013; Filho et al. 2009). Thus, this in vitro study used the indirect measurement method.

It would have been conceivable to scan the impression with a model scanner directly and create a digital model based on the scan. This approach would have allowed separate evaluation of the conventional implant impression. However, this would have introduced an additional measurement system, making the results difficult to compare. A drawback of the indirect measurement method is that it captures not only the dimensional changes of the impressions but also potential undesired changes resulting from the creation of the gypsum models. This inherent material error source was minimized by adhering to manufacturer guidelines and maintaining standardized laboratory conditions. Model measurements in this study were conducted using Final Surface software (GFal, Berlin, Germany). The master models were first aligned with the reference model using the modified best‐fit method, followed by distance analysis at the scan bodies. The average values calculated from the deviations of the three measurement points for each scan body were then compared. The best‐fit superimposition of the collected objects with the reference object can be performed without a fixed coordinate system. Two data sets are aligned, and an algorithm calculates the smallest possible distance between all defined points, effectively accounting for clinically relevant torsions and distortions (Persson et al. 2008).

Alternatively, linear distance measurement could have been used, as it is widely applied in numerous studies and has become established for assessing the transfer accuracy of impressions (Simeone et al. 2011; Lim et al. 2018; Kim et al. 2015). However, studies by Chandran et al. (2010) and Luthardt (2003) found that pure distance measurement between two points does not capture all dimensional changes and that a three‐dimensional analysis provides more precise information (Chandran et al. 2010; Luthardt 2003). Therefore, this study used a three‐dimensional distance analysis as recommended.

The accuracy of implant impressions has been the subject of numerous scientific studies, focusing on the influence of impression techniques, materials, and trays, as well as the accuracy of conventional and digital implant impressions (Nakhaei et al. 2015; Lee et al. 2008; Gallucci 2011; Del'Acqua et al. 2010). A product‐specific investigation of transfer accuracy, as conducted in this in vitro study, is not currently available in the literature. The results of this study indicate that the transfer accuracy of the closed‐tray technique among the five examined systems showed no significant differences. The comparison with the “SIC Invent open” system also revealed no significant differences in precision. Several studies corroborate that impressions using the repositioning technique achieve comparable transfer accuracy to the pick‐up technique (Lee et al. 2008; Gallucci 2011; Del'Acqua et al. 2010). A review by Lee et al. (2008) found that seven out of 14 analyzed studies showed no significant differences in transfer accuracy between open and closed implant impressions. Two studies favored the repositioning technique for higher transfer accuracy, whereas five studies found the pick‐up technique to be more precise (Lee et al. 2008).

The results of this in vitro study are challenging to compare with those in the literature due to different underlying measurement methods. Although many superimposition software systems report deviations using the root mean square error (RMSE), the present study deliberately focused on the mean deviation values derived from three clinically relevant measurement points per scan body (occlusal, buccal, and mesio‐distal), reflecting implant‐axis–related positional accuracy relevant for prosthetic fit. This approach enables localized assessment of transfer discrepancies at defined implant‐related landmarks. RMSE values, although suitable for global surface comparison, may obscure localized positional deviations that are clinically relevant in implant prosthodontics. However, across five commercially available implant systems (SIC Invent, BEGO, Bredent, Camlog, and Straumann), closed‐tray impressions demonstrated mean deviations ranging from 0.02 to 0.14 mm—well within clinically acceptable thresholds—and did not differ significantly from the open‐tray comparator (0.05–0.19 mm). The occurrence of isolated outliers across all evaluated systems suggests random procedural or material‐related influences rather than technique‐ or manufacturer‐specific inaccuracies. Potential contributing factors may include minor variations in transfer cap seating, impression material distortion, or cast fabrication processes. As these deviations were sporadic and not systemically clustered, they did not affect the overall comparative conclusions of the study. These data substantiate that modern transfer‐cap protocols can reliably reproduce three‐dimensional implant positions, irrespective of manufacturer, and achieve precision on par with pick‐up methods. However, this study has several limitations. As an in vitro investigation, it cannot fully replicate clinical conditions such as saliva, patient movement, intraoral temperature variations, or soft tissue resilience. In addition, the sample size was limited, and minor deviations from normal data distribution were observed. Only one impression material and a standardized laboratory workflow were evaluated. Consequently, the findings should be interpreted as indicative rather than definitive, and further clinical studies are required to confirm these results under in vivo conditions.

## 5 Conclusion

Within its limitations, this in vitro study demonstrates that closed‐tray implant impression systems utilizing transfer caps can achieve high levels of accuracy, comparable to traditional open‐tray methods. No statistically significant differences in transfer precision were observed among the five closed systems tested or when compared to the open‐tray (pick‐up) technique. The results of this investigation partially align with values reported in the literature. Various factors, such as the choice of implant system, impression technique, impression material, or tray, can influence transfer accuracy in implant impressions. Future studies should therefore incorporate clinical conditions to further validate these findings.

## Author Contributions

All authors have made substantial contributions to the presented work, have approved the submitted version, and agree to be personally accountable their own contributions. Conceptualization. Benedikt Schneider and Walter Zweigardt. Methodology. Walter Zweigardt and Benedikt Schneider. Validation. Constantin von See and Richard Mosch. Formal analysis. Dragan Ströbele and Richard Mosch. Investigation. Walter Zweigardt and Benedikt Schneider. Resources. Richard Mosch and Dragan Ströbele. Data Curation. Walter Zweigardt and Dragan Ströbele. Writing – original draft preparation. Walter Zweigardt and Benedikt Schneider. Writing – review and Editing. Constantin von See. Visualization. Benedikt Schneider and Walter Zweigardt. Supervision. Constantin von See. Project Administration. Constantin von See.

## Funding

The authors have nothing to report.

## Conflicts of Interest

The authors declare no conflicts of interest.

## Data Availability

The data that support the findings of this study are available from the corresponding author upon reasonable request. The raw data supporting the conclusions of this article will be made available by the authors on request.
